# Tumor-to-Tumor Metastasis: Lung Typical Carcinoid Metastatic to Follicular Variant of Papillary Thyroid Carcinoma

**DOI:** 10.1007/s12022-022-09706-4

**Published:** 2022-02-09

**Authors:** Ugolini Clara, Del Frate Rossella, Rossi Giulio, Materazzi Gabriele, LiVolsi Virginia

**Affiliations:** 1 Department of Surgical, Medical, Molecular Pathology and Critical Area, University of Pisa, via Roma 67, 56100 Pisa, Italy; 2 Department of Operative Unit of Pathology, Azienda USL Romagna, Infermi Hospital of Rimini, Rimini, Italy; 3 Department of Pathology and Laboratory Medicine, University of Pennsylvania Perelmann School of Medicine, Philadelphia, PA USA

**Keywords:** Tumor-to-tumor metastasis, Lung typical carcinoid, Thyroid, Papillary thyroid carcinoma, Follicular variant, Neuroendocrine tumor

## Case Presentation

In August 2020, a 70-year-old man presented with a rapidly growing nodule of the left thyroid lobe and was referred for a total thyroidectomy.

**Fig. 1:**
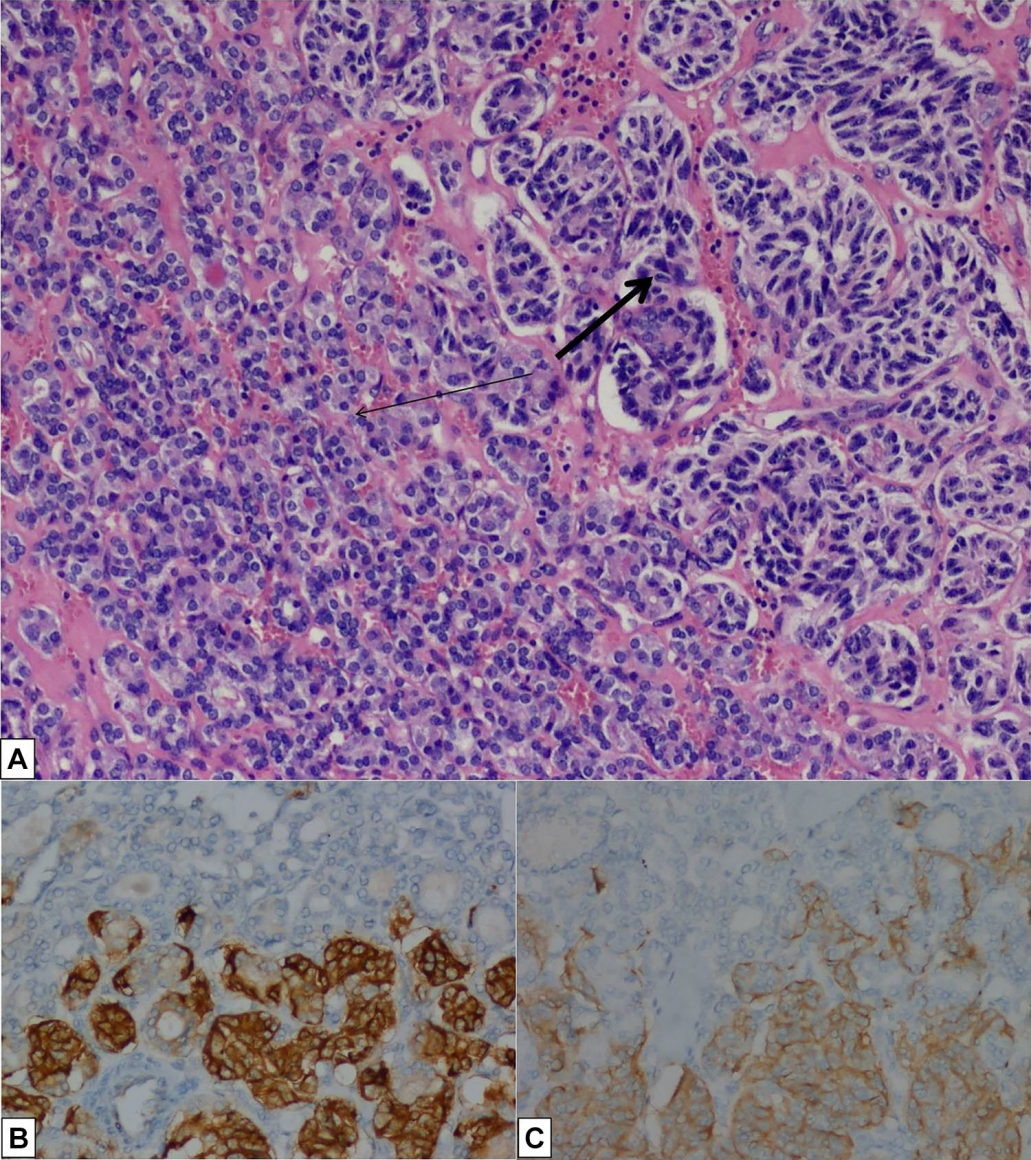
**A** Neuroendocrine tumor metastasis (bold arrow) inside papillary thyroid carcinoma (thin arrow) (10× magnification); **B** Chromogranin expression in the neuroendocrine component (20× magnification); **C** Synaptophysin expression in the neuroendocrine component (20× magnification). Other biomakers are not illustrated in this composite figure

The histological examination revealed a neoplasm with two different admixed components: an encapsulated follicular variant of papillary thyroid carcinoma (FVPTC) and a more internal component with neuroendocrine appearance. The FVPTC showed follicular growth and characteristic nuclear features of papillary carcinoma. The neuroendocrine component demonstrated uniform cells, with elongated shapes and finely granular chromatin, inconspicuous nucleoli, and a moderate amount of cytoplasm; it had a solid and trabecular growth pattern. Both the components showed tumoral capule infiltrations. Immunostaining showed that the neuroendocrine component stained for chromogranin A. Conversely, thyroglobulin, TTF-1, and PAX-8 were positive in the papillary carcinoma component (Fig. 1). Morphological and immunophenotypical evidence pointed to a neuroendocrine neoplasm which metastasized to FVPTC.

At this time, the clinical history of the patient became known to us. In 2007, the patient was diagnosed with a peripheral typical lung carcinoid (LC). In January 2010, he underwent a laminectomy D5-D6, revealing a metastatic pulmonary neuroendocrine tumor (NET).

## Discussion

Metastases to the thyroid gland are rare events. The most frequent tumor metastasis to the thyroid is carcinomas of the kidney, lung, colon and breast, and melanoma while malignancies from other sites rarely involve the gland [1, 2].

A rarer event involves the presence of tumor-to-tumor metastasis in the thyroid. Strict criteria are required to accept such a case: the recipient tumor must be a true neoplasm, and the donor tumor must be a tumor metastasis (direct contiguous spreading is not sufficient to define a tumor-to-tumor metastasis) [3].

Prior reports have described neuroendocrine tumors metastasizing into the thyroid gland. The first article was possibly reported by Laguette et al. who reported six cases, the majority of which derived from primaries in the lung, while only one possessing the morphological features of a typical carcinoid [4]. In 1999, Baloch et al. described three neuroendocrine tumors metastasizing into FVPTC, with primaries from lung, pancreas, and kidney [5].

In general, reported cases of lung NETs metastatic to the thyroid have been of intermediate-grade NETs (“atypical carcinoid”) or neuroendocrine carcinomas. Specifically, the combination of marked nuclear atypia, high mitotic rate, and necrosis was the criterion used to categorize the cases as atypical carcinoid tumors.

In these cases, selective studies of immunohistochemical markers, including thyroid markers, such as thyroglobulin and TTF-1, and molecular markers are necessary for differential diagnosis. Primary thyroid tumors with neuroendocrine features including MTC or mixed medullary-papillary carcinomas need to be considered and excluded.

Our case showed positive expression for thyroglobulin and TTF-1 in the papillary component while the LC component showed positive expression for chromogranin A and negativity for TTF-1, thyroglobulin, PAX-8, m-CEA, and calcitonin. This immunohistochemical pattern excluded both MTC and mixed thyroid carcinoma.

To further support our hypothesis, we have separately investigated the mutational status of HRAS proto-oncogene, GTPase (*HRAS*), KRAS proto-oncogene, GTPase (*KRAS*), NRAS proto-oncogene, GTPase (*NRAS*) and B-Raf proto-oncogene, and serine/threonine kinase (*BRAF*) genes with different techniques. The FVPTC was positive for the mutation c.181C > A (p.Q61K) in *HRAS* gene, whereas sequencing analysis of the neuroendocrine component did not reveal any mutation.

In summary, this case shows an occurrence of tumor-to-tumor metastasis of a typical carcinoid of the lung involving a FVPTC. Although exceedingly rare, secondary tumors of the thyroid gland should always be especially considered in the clinical setting of a previous malignancy.
