# Correction to: Tumor-to-Tumor Metastasis: Lung Typical Carcinoid Metastatic to Follicular Variant of Papillary Thyroid Carcinoma

**DOI:** 10.1007/s12022-022-09717-1

**Published:** 2022-05-06

**Authors:** Ugolini Clara, Del Frate Rossella, Rossi Giulio, Materazzi Gabriele, LiVolsi Virginia

**Affiliations:** 1 Department of Surgical, Medical, Molecular Pathology and Critical Area, University of Pisa, via Roma 67, 56100 Pisa, Italy; 2 Department of Operative Unit of Pathology, Azienda USL Romagna, Infermi Hospital of Rimini, Rimini, Italy

## Correction to: Endocrine Pathology https://doi.org/10.1007/s12022-022-09706-4

Figure 1 caption in the original publication contains a mistake.

The original article has been corrected.

